# Norwegian “Digital Border Defense” and Competence for the Unforeseen: A Grounded Theory Approach

**DOI:** 10.3389/fpsyg.2018.00555

**Published:** 2018-04-19

**Authors:** Ole Boe, Glenn-Egil Torgersen

**Affiliations:** Norwegian Defence University College, Oslo, Norway

**Keywords:** Strategic Competence Leadership, Cyber Defence, leadership, the unforeseen, military personnel, cyber-attacks

## Abstract

Strategic Competence Leadership (SCL) is a tool meant to ensure that the Norwegian Armed Forces Cyber Defence (NAFCD) has the necessary expertise to cope with current and future unforeseen events. This is necessary in order to develop a better digital border defense against digital threats from different state or non-state actors. Unforeseen cyber incidents include for instance “zero-days” attacks and similar severe threats. The goal of the SCL in the NAFCD is the development of a reliable “Cyber Resilience.” In the present study, we examine how the concept of SCL is understood and carried out on the different leadership and managerial levels in the NAFCD. Our research problem was how well prepared the NAFCD is for SCL and competence development for future competence needs, especially in relation to unforeseen events? Semi-structured interviews and document analyses of governance documents were used in the present study. The Strategic Didactic Model for the Unforeseen was used to construct nine research questions that were then given to the participants. The nine questions were sorted under three categories: organizational structures, competence development and needs, and plans, handling and communication. Fourteen leaders at different levels in the NAFCD participated in the study. Our main finding was that high-level (*n* = 4) and low-level (*n* = 10) participants differed in their views on SCL. As for unforeseen events, both levels shared the opinion that the NAFCD can cope with a low level of didactical degrees of the unforeseen (the degree to which the management has the competence to facilitate for adequate exercises for the different hierarchical levels in the organization, i.e., sudden unknown cyber-attacks). However, no common understanding of the term SCL was found among the participants. Interaction was interpreted to be very important to the participants, and they used professional networks to a high degree. In addition, unofficial lines of communication were very frequently used in relation to human resource exchange and development. By creating a common understanding within the NAFCD regarding the concept of SCL, it will be possible to improve the Norwegian digital border defense against unforeseen events.

## Introduction

Our purpose in this article has been to investigate whether the Norwegian Armed Forces Cyber Defence (NAFCD) has what it takes in light of Strategic Competence Leadership (SCL) to develop a robust “digital border defense." A digital border defense simply means that one has the ability to defend against different digital threats before they enter into one’s national territory (Forsvaret, 2018).

Most of the digital traffic in and out of Norway goes through high-speed fiber connections. This may render Norway susceptible to different types of digital threats. Examples of types of digital threats can be complex computer attacks that will affect the society at large and Norway’s defense capabilities, or communication between terrorists planning attacks in Norway (Norway Today, 2018).

There are political expectations that the Norwegian Armed Forces (NAF) must become a modern competence organization (Heier, 2017). In recent years, the road to this goal has passed through stricter requirements for so-called “Strategic Competence Leadership” (SCL). A definition of SCL in the NAF is: “The defense sector’s continuous process for planning, implementation and evaluation of measures to ensure correct competence at the correct time so that we can solve the required assignments. The process is integrated into the sector’s overall assignments and strategies. Leaders at all levels are involved” (Forsvarsdepartementet, 2014, p. 3, authors’ translation). This is a good definition, but it is still possible to ask critical questions about what the NAF’s real competence portfolio is, what critical core competence is, which competence requirements must be set, and what future skills needs will be (Boe, 2017). However, a possible problem is that the answers to such questions may vary according to where the respondents are positioned in the organization (Allison, 1969). There also seem to exist a generational gap between junior personnel and higher-ranking officers indicating that different generations tend to look for different answers regarding the cyber domain (Roislien, 2015).

In this article, we therefore ask whether the NAFCD has what it takes in light of SCL to develop a robust “*digital border defense.*” Our research problem is:

How well prepared is the NAFCD for SCL and competence development for future competence needs, especially in relation to unforeseen events?

In this study, we emphasize a conceptual and theoretical approach based upon Glaser and Strauss grounded theory (1967). In addition, we used politics-guided governance documents related to SCL and that applies to the entire NAF. In summary, the present study thus reveals an educational-psychological status quo at the NAFCD in Norway, based on the data and sources we had available when conducting the study. This is important, as it is well known that humans have a tendency to continue or maintain their previous actions (Samuelson and Zeckhauser, 1988). It is also well known that the decisions one is about to make are anchored in previous decisions (Ritov and Baron, 1992). An investigation of the educational-psychological status quo within the NAFCD will describe the current mental state within the NAFCD. If the current mental state also reveals that the NAFCD prefers the current status, this might present a barrier to the NAFCD as leaders may prefer to stay with what they know. This may hamper the possibilities of developing new measures to deal with cyber-attacks and could make the digital border defense less effective.

### Competence Development

Competence development requires broad involvement, it requires that one has a direction for the development and that the new knowledge and competence is implemented into the organization. Competence development for its own sake may work against its purpose and may result in the employees ending up with the wrong competence for the tasks they are supposed to execute. Lai (2004) calls this *incongruence competence*, or incompetence, i.e., a mismatch between the organization’s requirements and needs and the competence the employees possess. This kind of badly planned competence development can lead to demotivated employees. These employees may feel they are not in a position to use what they know, while the organization at the same time has tied up huge resources in a competence development program that is not needed.

### Strategic Competence Leadership and Interaction

The Strategic Human Resource Management (SHRM) approach involves making decisions on how the design of, e.g., learning and development is based on the organization’s intentions and schedules (Armstrong, 2006, p. 115). As mentioned, the NAFCD faces multiple and complex tasks that require high levels of expertise that must be continuously updated. This places great demands on how the organization’s competence is safeguarded and developed. If not, the ability to ward off digital attacks against Norway will be weakened. Nythun (2016) has pointed out regarding the NAFCD in Norway, that the interaction within the organization could be better, and that the different units had no common understanding of the organization’s tasks. It was also concluded that the organizational structure in some areas was perceived as dysfunctional and that the command lines were not sufficiently clear (Nythun, 2016). In the present study, the term “interaction” is used in order to further build on existing research in the field of interaction in the NAFCD (Nythun, 2016). Therefore, we choose the following definition:

Interaction is an open and equal communication and development process between actors that complement each other’s competence and exchange competence. Interaction can be direct face-to-face or mediated by technology or by hand, and work toward common goals where the relationship between the actors is always based on trust, involvement, rationality and organizational knowledge (Torgersen and Steiro, 2009, p. 130, authors’ translation).

This means that education programs that are designed to enhance SCL can support an improved interaction between different actors, in our case the departments within the NAFCD. The new governing plans for SCL includes report 14 “Competence for a new era” (Forsvarsdepartementet, 2013), and this report take interaction seriously. Report 14 provide guidance for both interaction, competence development, and management of competence measures. The starting point is an understanding of SCL as a fundamental coherent principle, and as a tool for strengthening the interaction in the organization. The new governance plan emphasize that the different organizations must have sufficient competence and that it must be developed in line with new challenges (ibid.). Particularly important is that the organizations should try to predict the skills needed in order to be at the forefront of necessary development. This is especially true of the NAFCD, which must constantly acquire new technology systems that will have to work against for example hackers who are usually in the forefront of established technology and software development. The NAFCD must therefore be prepared and be sufficiently competent to cope with surprising and unforeseen events. The interaction between humans, the technology, and the organization (HTO) is here a crucial point.

In this regard, there is reason to emphasize the importance of a hierarchy for the plans being designed by the Norwegian authorities. The plans have different degrees of detail, from the more superior ones developed in the Norwegian Ministry of Defence, to the more concrete plans intended for a limited part of the NAF. Collaboration is facilitated if there is good agreement between plans regardless of the different hierarchical levels. This will make it easier to recognize and implement the intentions in the governing plans. Based on the conclusions drawn by Nythun (2016), we wanted to look more closely at the interaction in the NAFCD. Poor compliance between plans, the understanding of these, and practices in an organization can contribute to problems of interaction and failure to manage the unforeseen. For example, having detailed plans is of little help if the leadership levels have only a superficial understanding of a central leadership tool like the SCL. If that is the case, it will be very difficult to identify which skills will be needed in the future (Forsvarsdepartementet, 2013).

### The Norwegian Armed Forces Cyber Defence

The Norwegian Armed Forces Cyber Defence (NAFCD) is a relatively new organization in the NAF. It is a continuation of the NAFs information infrastructure. The establishment of the new organization and the new name *Cyberforsvaret* in Norwegian (abbreviated to CYFOR in Norwegian) was decided by the Norwegian Government in 2012. The NAFCD also became a separate branch of the NAF when it was inaugurated in 2012. The core focus for the NAFCD is technological development within information and communications technology (ICT). The NAFCD further develops and protects the NAFs computer systems and command and control systems (C2). (Forsvaret, 2017). The NAFCD is geographically dispersed, the competence areas are very varied and this also affects the challenges they face. Often, language and vocabulary varies within the organization, in fact, they are so different that there is a lack of common understanding of the tasks of the NAFCD (Nythun, 2016). That this lack may lead to a great variation in the interpretation of SCL is not unexpected.

The NAFCD consists of two departments (Forsvaret, 2017). CYFOR CKT develops and establishes information systems that the NAF need to communicate and control their forces in an operating area. The focus areas of the CYFOR CKT department are the NAF’s reinforced focus on military operations within a joint operational framework, and the need for C2 systems that work across the various branches of the NAF (Forsvaret, 2017). CYFOR CTO is a military organization for the future. The departments’ primary tasks are delivery of services, operations, and defense of the NAFs ICT systems, as well as delivering sensor and radar data to operational environments. The departments are also responsible for technical operation and the further development of the NAFs integrated management system (abbreviated FIF in Norwegian). The departments contribute to community security through surveillance and information retrieval, and by providing infrastructure and mission critical services to key parts of the government administration (Forsvaret, 2017).

### Cyber-Attacks and Possible Challenges

The next section will describe cyber-attacks and some possible challenges that these attacks may lead to. Russian hacker attacks against Western institutions and political processes have come to the forefront lately (Gerodimos et al., 2017). A Google search with the words “Russian hacker attacks against Western institutions” results in approximately 570,000 hits. Although the attacks have grown in scale in recent years, the phenomenon is older. A well-known example is how targeted cyber weapons were used to attack and destroy Iranian weapons production facilities for nuclear weapons in 2010 (Anderson, 2012; Sanger, 2013). In the documentary film of the attack, *Zero Days*, it is emphasized how resource intensive and resource demanding it is to design a cyber weapon (Gibney, 2016). The documentary deals with the importance of having the ability to handle the unforeseen: the documentary states that no systems are safe, and that the best attacks are those that the opponent does not notice, as in a cyber-attack. The cyber domain is not regulated by conventions like nuclear weapons, and therefore, it becomes very difficult to control what is taking place within the cyber domain. It is therefore of vital concern to understand the cybersecurity challenges, and also to understand the essential components that constitute cyber operations (Buchanan, 2016).

The cyber-attack against the Iranian weapons production facilities was quite advanced. A malware was tailored to remove the centrifuges for enrichment of uranium from the production line (Sanger, 2013). However, the attack was carried out several years ago, and progress has been rapid since then. The cyber-attacks of today and of the future are often more sophisticated and the problems of identifying who are behind the attacks can be very difficult. Military attacks in the physical domain are thus far easier, they also make scenario thinking and scenario planning a more affordable task compared to cyber-attacks. The problematic point regarding cyber-attacks is that it is possible to be exposed to an extensive attack without ever detecting it (Lindsay, 2013). Deibert et al. (2012) have analyzed the impact of cyberspace on the conflict between Russia and Georgia in 2008. They refer to cyber warfare and discuss different issues that need to be dealt with regarding future comparative research. Among these are the importance of control over the physical infrastructure of cyberspace, the strategic and tactical importance of information denial, the emergence of cyber privateering, the unavoidable internationalization of cyber conflicts, and the tendency toward magnifying unanticipated outcomes in cyber conflicts. These issues are also important to deal with for the NAFCD in order to obtain a digital border defense that is functional.

### The Cyber World: A Lawless World?

Another challenge is that the cyber domain is barely subject to any laws or conventions, and this may pose a challenge for those who are being attacked. This therefore poses severe challenges to understanding how sophisticated attacks actually can be and where they can hit. Therefore, the staff of the organizations dealing with cyber-attacks must be trained to respond to the unforeseen. This means, for example, that they must quickly see that something is wrong even if the system itself indicates that everything is okay. They need to have the competence to be able to use preventive measures on a regular basis. In addition to this and further blurring the picture of cyber operations and cyber-attacks, there seems to exist a questionable moral within the cyber domain when it comes to conducting cyber-attacks. It has been stated that as long as a cyber-attack is not noticed by the actor, organization, or state being attacked, it is considered legal (Gibney, 2016). This attitude also contributes to blurring the morality of cyber-attacks.

### Cyber Operations and Central Concepts in the Norwegian Armed Forces

The following section will describe cyber operations and central concepts in the NAF. “The cyber dimension” is a concept that often is used to describe the digital space, generated by computers and networks, where cyber operations take place (NATO Review Magazine, 2011; Forsvarsstaben, 2014). Cyber operations are a term used synonymously with “Computer Network Operations” (CNO) in the Norwegian Defence Sector (Forsvarsstaben, 2014). Cyber operations are thus measures implemented in computer networks to affect the opponent’s data networks and protect one’s own networks. This includes Computer Network Defence (CND), Computer Network Exploitation (CNE) and Computer Network Attack (CNA). In Norway, responsibility for the cyber operations within the NAF is shared between the Norwegian Intelligence Service (NIS) and the NAFCD (Forsvarsstaben, 2014). These departments cooperate with the Norwegian National Security Authority (NSM in Norwegian), which is also part of the Norwegian Defence Sector. The NSM has a national coordinating responsibility for the protection of information and infrastructure of importance for societal functions. The NSM also has a national cross-sectoral responsibility for identifying serious cyber events, notifying relevant actors, and coordinating the handling of the events. Furthermore, individual companies and organizations have an independent responsibility for building barriers against cyber-attacks. However, attacks with terrorist motives or other criminal motives could affect both directly and indirectly both state and private organizations as well as individuals (Peters, 2005). The current chief of the NAFCD, Major General Inge Kampenes, also expresses concern about the somewhat unclear division of responsibilities between the various organizations (Forsvarets Forum, 2017).

So-called “defensive cyber operations” will support military operations and strategic crisis management, and in armed conflicts will be part of the overall operational planning and included in “information operations.” Through Norway’s participation in NATO and/or Allied Operations, the coordination of cyber resources will be included as part of the NAF overall operational coordination with these stakeholders (Aftenposten, 2017). Defensive cyber operations include both monitoring and handling cyber-attacks, to reveal reconnaissance, infiltration and attacks as early as possible.

The NAF also has capacities for “offensive cyber operations.” The main purpose of these capacities is protecting the digital systems, and possibly other infrastructure, from attacks. Recognition and detection of threats are central means. However, experience shows that cyber-attacks may include new and unforeseen methods and software. Therefore, cyber training for the unforeseen will be an important method in both defensive and offensive cyber operations (Torgersen, 2015).

### The Unforeseen

“War is for participants a test of character. It makes bad men worse and good men better” (Chamberlain, 1915, p. 295).

The following section will describe the concept of the unforeseen, as well as related concepts and a training model for the unforeseen. A common theme for military forces around the world is the changes, uncertainty, complexity, and ambiguity that characterize many modern military operational environments. These situations are also referred to as VUCA situations, an acronym used to describe the volatility, uncertainty, complexity and ambiguity of different conditions and situations (Stiehm, 2002). An additional challenge is “asymmetric” warfare, that is, a type of war characterized by terrorism, guerrilla warfare, and ideological manipulation (Matthews, 2014). Norwegian soldiers and officers have been participating in several operations in different countries with an increasingly difficult operational environment (Boe et al., 2012). This forces the military leader to constantly rethink his or her role, as well as their norms and values in the chosen military profession (Snider and Matthews, 2012). As a consequence, they also need to consider how they will be able to perform different duties and specific missions under extreme stress.

“The unforeseen denotes something that occurs relatively unexpectedly and with relatively low probability or predictability for those who experience and must deal with it” (Kvernbekk et al., 2015, p. 30, authors’ translation). In addition to the unforeseen, there are concepts such as the uncertain, the unexpected, the surprising, the unknown, the unpredictable, the unthinkable, the unlikely and the random (Kvernbekk et al., 2015). Defining the concepts makes it easier to describe a situation in more detail. The unforeseen is also graded on a scale from 0 to 4 on each of three pedagogical factors. These factors are familiarity, notification, and escalation (see **Table 1** below). The three factors are defined in degrees that together form a didactic degree of the unforeseen (DD-UN) (Torgersen, 2015, p. 328).

**Table 1:** Examples of different levels of DD-UN based upon three pedagogical overarching factors for the unforeseen.

	Pedagogical overarching factors for the unforeseen		
Leve of DD-UN	Familiarity	Notification	Escalation
0	Absolutely unknown	No preparation	No known warning sign
1	Some known characteristics	No preparation	Some known warning signs
2	Some known characteristics	Some preparation	Some known warning signs
3	Several known characteristics	Some preparation	Some known warning signs
4	Identical characteristics	Preparation	Several known warning signs

The concept unpredictable has been defined by Kvernbekk et al. (2015, p. 32, authors’ translation) as follows: “somewhat tangible, but you do not know when, where, how, or if it strikes, or against who or what. Actions without a fixed pattern.”. This concept is strongly linked to the unforeseen, and our participants, when explaining the unforeseen, mentioned among other things the unpredictable. **Table 1** shows the different levels of DD-UN based upon three pedagogical overarching factors for the unforeseen (Torgersen, 2015, p. 328). The overarching aim for any organization dealing with cyber-attacks should be to acquire the competence to deal with events that are absolutely unknown (familiarity), where there is no time for preparation (notification) and no known warning signs (escalation), known as DD-UN level 0. However, organizations may find that they only have the competence to deal with DD-UN levels 1–4 where they are less equipped to deal with unforeseen cyber-attacks.

In this article, we are concerned with how SCL is promoted and exercised in the NAFCD. The key here is how leaders and managers at different levels of the organization perceive SCL. The governing document report 14 is forward-looking and provides political guidance for the direction of the NAFs development (Forsvarsdepartementet, 2013). In this regard, we were interested in the level of competence to master unforeseen events involving humans, technology and organization (HTO).

### The Strategic Didactic Model for the Unforeseen

**FIGURE 1:**
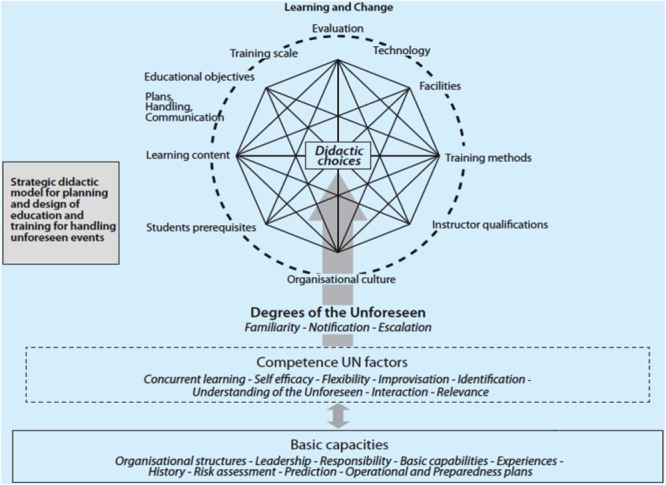
The Strategic Didactic Model for the Unforeseen (SD-UN) (modified after Torgersen, 2015, p. 330).

Hollnagel (2003, 2009) is concerned with the organizational framework of the interaction between humans and technology (HTO). He is particularly concerned with the employees’ ability to quickly recover systems when errors occur, and with to what extent planning takes this into account. This resembles the well-known leadership theorist Argyris (1992) introduction of the concepts of single-loop and double-loop learning. There is a significant difference in complexity between the two: “Single-loop learning is appropriate for the routine, repetitive issue – it helps get the everyday job done. Double-loop learning is more relevant for the complex, non-programmable issues” (p. 9). Another important difference is how the organization responds to problems and errors that are discovered along the way in the learning process. Single-time learning occurs when the difference between the present situation and the desired situation is smoothed by using corrective actions (ibid.). An example of single-loop learning is a unit that finds it is lacking a form of competence to solve a specific assignment and that then sends personnel to acquire the needed competence in order to cover the shortfall. Double-loop learning occurs when mismatches are corrected after the problems have been analyzed, variables have been changed and corrective measures have been implemented (ibid.). For our research purposes, it is the double-loop learning which is important in determining how SCL is taken care of in the NAFCD. However, the NAFCD is an organization that works both with routines and unforeseen events, so both simple and double-loop learning should be present. By using the SD-UN model (see **Figure 1**), one can grade the unforeseen into different levels. And this would for example identify the operational requirements and needs that will make it possible to map the skills needed to reach them.

Torgersen (2015) writes about flexible organizations and proposes a Strategic Didactic Model for the Unforeseen (SD-UN). The SD-UN model is a framework for linking different theories together (Tennyson and Foshay, 2000; Mitroff et al., 2004; Torgersen and Sæverot, 2015). The model consists of four main elements: learning and change, degrees of the unforeseen, competence unforeseen factors, and basic capacities. Based upon the SD-UN model, we developed nine questions to be used in the present study. **Figure 1** below gives an overview of the SD-UN model.

**FIGURE 2:**
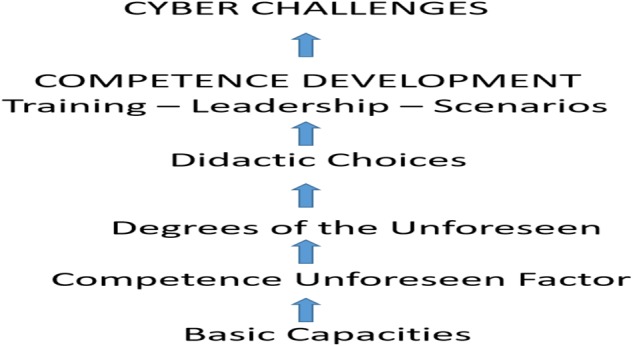
A training model for cyber competence and the unforeseen.

By using the SD-UN model, one can grade the unforeseen into different levels. When one has a degree of the unforeseen, it is possible to define for instance ambition levels regarding preparedness, training and exercises, and construction of scenarios. The SD-UN model can also be used to design questions about competence unforeseen factors and basic capacities. Factors from the competence unforeseen factors element such as cognitive flexibility (Spiro et al., 1992), improvisation, identification (of warning signs), the unforeseen concept itself, and interaction are unforeseen relationships that will be highlighted in the present study. Based upon the SD-UN model, a training model for cyber competence and the unforeseen can be developed. **Figure 2** shows the components of the DD-UN as a training model for cyber competence and the unforeseen.

The conceptual thinking of the training model is that in order to meet and cope with cyber challenges, one has to first have some basic capacities. Dependent upon the levels of competence unforeseen factors and the different levels of the unforeseen, different didactic choices will have to be made. This will then produce and result in different competence development within training, leadership and scenarios, with the aim of being able to meet unforeseen cyber challenges.

Several organizational theorists have mainly based their research on companies or public organizations where the technological component is not particularly advanced (Argyris, 1992; Pfeffer and Sutton, 2000; Armstrong, 2006). Two researchers who have used the same perspectives on organization’ ability to resist cyber-attacks are Herrington and Aldrich (2013). They use the term “Cyber-Resilience.” A definition of Cyber-Resilience is the ability of a network to continuously deliver the intended outcome despite adverse cyber events (Björck et al., 2015). One of Herrington and Aldrich (2013) findings is that the less sophisticated and integrated the cyber domain is in society, the smaller the consequences of cyber-attacks. Achieving Cyber-Resilience requires the ability to control infrastructure analogously or with personnel that can push buttons and read instruments manually (Herrington and Aldrich, 2013). In the latter case, it will require personnel that have the competence needed to run the systems both through the cyber domain and manually where the equipment is positioned. Guy Benveniste (1994) emphasized that as systems become more complicated, there is less room for operators to control and take care of the equipment. Other contributors in this field are Kenney (2015) and Lindsay (2013), who both discuss what kind of cyber-attacks are most likely, based upon the different actors’ motivation and capabilities to conduct attacks.

A researcher who looks closely at the importance of regular routines to handle unforeseen events is Sheffi (2015). Sheffi is concerned with the reaction time from a crisis being detected until action is taken and normal mode is restored. One conclusion drawn is that fixed structures and standards can provide speed and flexibility as well as stamina and rigidity. By providing well-trained people with confidence and the authority to adapt the training to handle new situations one can achieve flexibility and speed when new situations occur (Sheffi, 2015).

### Aims of the Present Study and the Research Problem

Based upon the previous discussion of the challenges facing the NAFCD, the aim of the present study was to investigate the following research problem:

How well prepared is the NAFCD for SCL and competence development for future competence needs, especially in relation to unforeseen events?

In particular, we wanted to investigate what level of unforeseen events the NAFCD can handle. Stated somewhat differently, the personnel must be able to handle situations that occur suddenly and surprisingly, with an unknown content, where outcomes of actions are characterized by low degree of predictability (i.e., the unforeseen) (Torgersen et al., 2013). Facing these types of situations often requires a type of leadership commonly referred to as “in extremis leadership” (Kolditz, 2010).

## Materials and Methods

### Data Collection

Parts of the dataset used in this study has previously been described in a master thesis at the Norwegian Defence University College (Johansen, 2017). We have used semi-structured interviews and document analysis by looking at the Norwegian governing documents dealing with SCL.

### Document Analysis

The most important documents used in the document analysis have been the Norwegian government report 14 “Competence for a new era” (Forsvarsdepartementet, 2013), the “Human Resources Directives in NAF” (Forsvarssjefen, 2014), the “Norwegian Defence Sector Basic Values” (Forsvarsdepartementet, 2012), and the pre-study of the personnel and competence in the defense sector (Forsvarsdepartementet, 2011). Additional documents used in the analysis have been the “Norwegian Armed Forces HR Strategy” (Forsvarssjefen, 2015a) and the “Norwegian Armed Forces basic values” (Forsvarssjefen, 2015b).

As far as it has been possible to determine, the NAF did not issue other key documents that took into account SCL during the time the present study was conducted.

### Participants

In the present study, we choose to describe the persons being studied as participants. This is in accordance with Morse (1991) who postulates that the term participants symbolize a more active engagement from the persons being studied, and that the term is commonly used in qualitative research.

In order to ensure that the selection of participants was representative, i.e., that the breadth of opinions and perceptions of SCL in the NAFCD would appear in the study, we arrived at the following selection criteria: leaders from both the CKT and the CTO departments of the NAFCD would have to be represented; responses would have to come from leaders at all levels; the proportion of female participants would be in minority, but their numbers should not differ significantly in percentages from the total number of female leaders in the NAF. We initially wanted to include five managers per level of leadership to ensure that the collected data had an adequate size. The interview process started out with just a few participants, additional names were provided with the help of the already selected people. Thus, the selection could be described as a convenience sample (Morse, 2007).

Due to challenges with finding enough leaders that were willing to participate in the study, we ended up with fewer participants than we planned for. The end result was that we thus selected participants on the basis of a grounded theory approach (Glaser and Strauss, 1967; Starrin et al., 1997). In our case this meant that our ambition was to obtain a wide variety of experiences and hierarchical levels from leaders at different levels in the NAFCD.

**Table 2** gives an overview of the number of participants who participated in the study, the number of women in the study and also which data collection methods that were used at the different levels of leadership. The participants were divided into two group, one consisting of the higher-level leaders, and one group consisting of lower-level leaders. High-level participants refer to section leaders and above in the NAFCD. Low-level participants indicates employees who are subject matter experts and technology experts positioned lower in the hierarchy. We chose not to define the groups any further, because of the participants possibility to remain anonymous and the few number of participants in total.

**Table 2:** An overview of interviews according to leadership levels, number of participants on each level, and data collection methods used at different leadership levels (*n* = 14).

	*n*	*k*	Data collection methods used
High-level group	4	0	Semi-structured interviews
Low-level group	8	2	Semi-structured interviews

*n, number of male participants; k, number of female participants*.

The reason for dividing the participants into two groups was to investigate whether there existed any differences in the understanding of SCL and in the ability to cope with unforeseen incidents. As we had only two female participants, we decided to include them in our analyses and not to use gender as a variable.

### Procedure

An application was sent to the commander of the NAFCD in order to gain permission to complete the study. A permission was granted to conduct the study. As we wanted to conduct audio recording of the interviews for later transcripts, an application was also sent to the Norwegian Social Science Data Service (NSD) to gain approval for the study. The study was approved by the NSD and written informed consent was obtained from the participants of the study.

After identifying potential participants, an e-mail was sent out. It contained an information letter about the study and why the recipient was in the target group. The recipients who did not answer were followed up by a text or phone call a few weeks later to ascertain whether they would participate. Eventually, a schedule was set for when the interviews were to be conducted. Up to 1.5 h was planned for individual interviews. The individual interviews lasted between 40 min and 2 h.

### Data Collection

We conducted qualitative semi-structured interviews following a prepared interview guide to collect the data. The interview guide had previously been tested in a couple of pilot interviews. The interviews consisted of nine open-ended questions. They followed a chronological structure with one question related to each of the two didactic choices organizational culture and evaluation found in the element learning and change in the SD-UN model. Thereafter, questions related to competence development and needs, derived from the element competence unforeseen factors in the SD-UN model were asked. Finally, questions related to the didactic choice plans, handling, and communication found in the learning and change element in the SD-UN model was asked. We ended up with three overall categories of answers, organizational culture, competence development and needs, and plans, handling and communication. Two categories thus belonged to the element learning and change and one category belonged to the element competence unforeseen factors in the SD-UN model. The open-ended questions and categories were derived from possible questions that we felt we would need to ask the participants in order to answer our research problem. **Table 3** gives an overview of the open-ended questions and categories used in the semi-structured interviews.

**Table 3:** Questions asked to the participants in the semi-structured interviews.

**Category learning and change: Organizational culture and evaluation**
(1) What in the framework of the organization do you think limit the implementation of the competence development necessary for your department to better fulfill its mission?
(2) How are courses and training for the staff in your department evaluated?
**Category competence unforeseen factors: Competence development and needs**
(3) Which competence development measures does the NAFCD have?
(4) What are the differences in the content and how the appraisal interview is conducted for civilian and military staff?
(5) How are new competence needs identified?
**Category learning and change: Plans, handling, and communication**
(6) How is the competence development for the employees planned?
(7) What competence is planned?
(8) Is there any overall plan for what competence your department should have?
(9) Which leads come from a higher level regarding the priorities of competence?

As can be seen from **Table 3**, we asked our participants nine questions that would answer the research question of how well prepared the NAFCD was for SCL and competence development for future competence needs, especially in relation to unforeseen events.

### Validity

The question of transferability or generalization of our data boils down to whether the analyses and the findings can be used in similar and appropriate situations (Lincoln and Guba, 1985). The NAFCD is a comparatively small branch within the NAF, and therefore, we cannot generalize our results. Also, the number of participants in our study was quite limited. However, our results give a glimpse of the current status within the NAFCD regarding the perceived level of SCL at different leadership levels, as well as the perception of competence development for future competence needs, especially in relation to the unforeseen.

### Data Analysis

The interviews were first transcribed verbatim and were later analyzed consecutively according to the constant comparative method (Glaser and Strauss, 1967). The first step in this method is known as “open coding.” This means that data are examined line by line to identify the participants’ descriptions of actions related to the themes mentioned in the interviews, as well as thought patterns and feelings associated with these actions. After that, codes are formulated in words resembling those used by the participants. To illustrate:

“For example, when we have we been handed a new tool, and then there is no money for competence development for those who actually work with it and have to live with it every day.”

This excerpt was coded as “*attitudes toward maintenance personnel*.” The second step was to sort the codes into one of the nine different questions posed in the interviews. The previous excerpt was sorted under the question “*What in the framework of the organization do you think limit the implementation of the skills development necessary for your department to better fulfill its mission*?”. This process of analysis was conducted by making constant comparisons between the interview transcriptions, codes and questions. Also, both the codes and questions were analyzed in respect to our selection criteria. This meant that comparisons were made between the different participants and their positions in terms of their hierarchical level.

For all practical purposes, our analytical steps were not strictly sequential. We constantly moved back and forth in re-examining the interview transcripts, codes, and categories. This corroborates well with a working procedure that is in line with the iterative process of the grounded theory method (Glaser and Strauss, 1967; Starrin et al., 1997).

## Results

The following sections will describe the answers the participants gave to the nine questions they were asked to ponder upon. First we describe the results from the questions related to organizational structures, then to competence development and needs, and finally to overall plans, handling and communication. In short, our main finding is that the low-level and high-level participants differed in their view on SCL.

### Organizational Culture and Evaluation

Two questions in the interviews dealt with the themes organizational culture and evaluation as to whether the NAFCD had the needed factors in place to be able to conduct competence development and to utilize the existing internal competencies.

#### Question 1: Limiting the Implementation of the Skills

The first question, *What in the framework of the organization do you think limit the implementation of the competence development necessary for your department to better fulfill its mission?* was asked during the interview, leading to the following answers:

Both low-level and high-level participants believed that economy and time were limiting organizational structures for competence development. Low-level participants also mentioned clarification of ambitions as a limiting organizational culture factor. Also, the prioritization of tasks was mentioned by low-level participants as limiting factor. Resources such as time and economy were also said to be used in an inappropriate manner. Furthermore, it was said from the high-level group that there was insufficient knowledge of the deliveries to the different branches of the NAF. Uncertainties about the level of notification and level of training also made it difficult to properly prioritize these factors within the organization.

Two of the low-level participants mentioned lack of implementation of skills as a limiting factor. “*When, for example, we have handed over a new tool and there is no money for competence development for those who actually work with and live with it every day.*” There was also a question of “*Policies for which education is accepted*.” A question that arises is whether the existing policies are good enough since they were mentioned as a limiting factor. It was also pointed out that the security clearance of individuals was too time consuming so that it took too long to fill the emergency needs. Lack of positions and people means that it will be harder to set aside time to work on competence development. Several participants emphasized that the rules for raising competence were too unclear when it came to military skills. Many stressed that the NAFCD’s many levels and geographical spread sometimes affected the transfer of experience between the employees in a negative way.

It was also found that the lack of an evaluation regime for the NAFCD’s services provided shortcomings. A high-level participant mentioned that this means that today it is a case of “letting the fox watch over the geese” in terms of control of how the services are delivered. This simply means that there is a risk that the NAFCD provides services to the branches of the NAF that are not adapted to the needs of the branches of the NAF. In addition, another high-level participant said that “*the other defense branches do not know the NAFCD well enough. We are there for the NAF, not for ourselves*.”

#### Question 2: Evaluation of Courses and Training for the Staff

The second question dealt with how courses and training for the staff was evaluated. Evaluation of courses and training is important. Otherwise one risks using the limited resources on poor development of competence. A thorough evaluation makes it possible to choose courses and training that are in line with expectations. In the interviews, the question asked was: *How are courses and training for the staff in your department evaluated?* The answers revealed that most participants did not undertake any systematic evaluation of courses or training. There were a few participants who answered that they evaluated all courses and travels with a report. Most participants answered that they received oral feedback on courses when the staff returned. Failure to evaluate courses and training for employees may cause employees to be sent on course and competence development without them actually being given the necessary skills. Torgersen (2015) claims that training for the unforeseen is an important method. However, the NAFCD seems to lack an awareness of evaluation training as a method in both defensive and offensive cyber operations.

### Competence Development and Needs

#### Question 3: Which Competence Development Measures Does the NAFCD Have?

Regarding the question “*Which competence development measures does the NAFCD have*?” we found that according to the participants, the NAFCD has a number of measures that can be implemented. The more traditional courses, education, and seminars were emphasized many times by the participants. Other measures like on-the-job training (OJT), professional conferences, discussions with professional specialists, and visits to other departments were also mentioned.

#### Question 4: Differences Between the Content and Conduct of the Appraisal Interview

The yearly appraisal interview is one of the tools to handle competence planning and competence mapping. Using the question *What are the differences between the content and how the appraisal interview is conducted for civilian and military staff*? it became possible to detect if there were any differences between military and civilian employees. Major differences could indicate a different focus on how the skills of the personnel are developed. However, according to our participants, there was almost no difference in how the appraisal interview was implemented. It also emerged that for military personnel, competencies that were required for advancement in the military system were discussed. A high-level participant stated: “*Next level competencies cannot be planned,… We can provide input, push, feed. They must take the initiative and implement it. We can make recommendations*.”

Competence development means that new competence needs are identified. An important aspect here is whether this is centrally controlled or locally initiated. In order for the organization to handle unforeseen changes and acquire competence, development should be initiated locally. On the other hand, a centrally controlled development provides opportunities to coordinate measures in order to reduce costs. Another advantage of centralized competence development is standardization so that staff can collaborate more easily across geographic areas and departments.

#### Question 5: Identifying New Competence Needs and Planning

The answers given to the question *How are new competence needs identified?* revealed that the requirements for renewal was a key driving force. The reasons for this were changes in development, materiel, and needs. The answers also show that participants have many ways to identify new skills needs. A high-level participant mentioned the strategic operational level. Here it may be a problem that the term SCL is linked to the strategic level in the military context. Another answer from a high-level participant was the use of expertise to reach the organization’s goals. This answer means that the term is no longer linked to a special level. High-level participants answered that new competence needs and planning was important in order to understand operational needs, to educate people, and for the strategic disposition of personnel. An overview of the skills needed in peace and in the rest of the conflict spectrum was also highlighted by our high-level participants.

### Plans, Handling, and Communication

#### Question 6: How Is the Competence Development for the Employees Planned

The question *How is the competence development for the employees planned?* revealed a distinction between low-level participants and high-level participants. The answers from the low-level group agreed in emphasizing the appraisal interview as an important tool. The results of the appraisal interview will find their way into the competence plan. In addition, the leader has a continuous dialog with the employees regarding competence development. Some courses are taken *ad hoc* when they become available or when employees report their interest. Participants in the high-level group said they had a plan that was flexible enough to handle the needs that arose. The answers that came from the high-level participants instead emphasized the operational needs and requirements that would form the basis for which competence the NAFCD should have. Here the participants were concerned with the operational requirements and needs that were not met. Overall, the answers from the high-level participants gave a less *ad hoc* impression of how the competence planning was conducted, compared to those of the low-level participants. Participants in the high-level group responded among other things that competence development was based upon an overall plan that contained a clear goal/objectives and means. The goals/objectives were linked to the activities of the NAFCD and overall goals/directives.

#### Question 7: What Competence Is Planned?

As a follow-up, the question: *What competence is planned?* was also asked to the participants. The answers were that it was essentially on technical competence that the attention was focused. In addition, purely military subjects and additional courses for the specialists were mentioned. Participants at the low level answered lack of long-term plans, lack of education, and lack of competence raising as reasons for personnel to leave the department. This raised the need to build the skills of the employees in the correct way.

#### Question 8: Is There an Overall Competence Plan

We asked the participants to answer *if there was any overall plan for what competence their department should have?*, in order to be able to predict what competence needs would arise in the future. The answers from the low-level participants pointed to the overall plan from which input was made, and the employee competence plans drawn up on the basis of the appraisal interview. In addition, assessments were made of what the department possessed in terms of expertise and what was missing. Answers from the high-level participants revealed that they were familiar with what the requirements were and what had to be done to fill them.

#### Question 9: Leads From Higher Levels Regarding the Priorities of Competence

In the long-term plans and budgets, one will find political guidance on the direction of the NAF’s development (Forsvarsdepartementet, 2013). This means that the requirements and assignments given to the branches of the NAF are sometimes changed. Such changes may also lead to the need for a change of competence in a department. It is therefore important that there is a certain dialog between the different levels when it comes to the prioritization of the competence to be developed. To find out if such priorities existed in the NAFCD, the following question was asked: *Which leads come from a higher level regarding the priorities of competence?*

The high-level participants got their priorities through the governance plan that was issued from the higher political level. The lower levels have been given the lead to prioritize “green training and soldier skills.” Green training and soldier skills here refers to military skills and abilities one acquires while in uniform. Examples are shooting, marching, and leadership training. Some of the low-level participants also mentioned courses that lead to health, environment, and security clearances, courses in FIF 3.0 (computer courses) and other FIF courses. A focus on “green training and soldier skills” might result in an inability to cope with higher levels of DD-UN, that is, level 0. This means that the familiarity with a cyber-attack is absolutely non-existent, there exists no preparation in relation to notification, and there is no known warning sign during escalation of the attack. Participants at the high level answered that they believed that the NAFCD can handle unforeseen events at high levels of DD-UN. One participant at high level believed that the NAFCD can handle unforeseen incidents at DD-UN at their own level, the other participants believed that the NAFCD was capable of only handling unforeseen events at DD-UN at level 1. In other words, senior leaders (i.e., high-level participants) believed that they could handle unforeseen events that are unknown with some known characteristics (familiarity), where there is no preparation time (notification) and with little or no warning signals (escalation) given in advance. This can interpreted as an ambitious attitude, and probably an over-estimation of their own expertise, in light of past experiences with cyber-attacks at international level, for instance the Stuxnet event (Anderson, 2012; Lindsay, 2013). An interpretation of the answers given to this question could be that senior leaders underestimate the different levels of DD-UN related to familiarity, notification, and escalation factors for the unforeseen. This may lead to the high-level leaders thinking they are at DD-UN level 0 or 1, whereas low-level participants may think they are at DD-UN level 2, 3, or 4. The challenge here is that at the higher DD-UN-level, that is, level 0 or 1, the more competence is needed in order to prevent, handle and to recover from unforeseen cyber-attacks.

Here, there also appears to be differences in how often the staff in NAFCD practice handling new unforeseen events. The high level believes that the staff are often trained in handling new unforeseen issues, while at the low level it is actually very rare that personnel are trained to handle unforeseen events. This means that the operational core of the organization perceives training plans to include more training on new unforeseen events than those who actually perform the training plans and who have the actual ICT skills.

## Discussion

A central finding in the research literature is that the most effective way of spreading expertise is through systematic dissemination. This happens by creating an environment for sharing knowledge (Chyi Lee and Yang, 2000). Whether this is taking place in the NAFCD is a question we sought to find the answers to. In this regard, it is important to look at how knowledge is transferred from individual to group and beyond, from group to organization (Nonaka, 1994; Nonaka and Konno, 1998). In this regard, Chyi Lee and Yang’s (2000) description of the knowledge value chain is relevant. They emphasize the dissemination side: that knowledge acquired by one person spreads to the others in the organization. This will happen if an environment is created for sharing knowledge. If this is done, an important prerequisite for SCL is fulfilled.

Our data reveals that one can see a difference in the way the concept of SCL is understood at the low-level and at high-level. The high-level has a more strategic thinking revolving around the term. At the same time, it appears that there is no unambiguous understanding of what SCL means in the NAFCD. However, some of the participants had a relatively similar understanding in that SCL has something to do with achieving overall goals and competence development. Here, this understanding is partly consistent with Armstrong’s (2006) understanding of SCL. Armstrong (2006) writes that SHRM (SCL in our terminology) is about making decisions about how the design of learning and development, among other things, is based on the organization’s intentions and plans (Armstrong, 2006). This definition makes it possible to compare goals and practices and whether the NAFCD attempts to implement SCL. Armstrong further states that the fundamental aim of SHRM is to generate strategic capability by ensuring that the organization has the skilled, committed, and well-motivated employees that it needs to achieve a sustained competitive advantage (Armstrong, 2006).

The organizational structure reveals a lot about how the internal learning of the organization is taken care of. An example is whether employees are able to work on their own skills enhancement. The annual appraisal interview is a strategic guide, for example, to planning competence development. These may be measures that are part of the SCL. Armstrong (2006) emphasizes that the human resource plays a strategic role in the organization’s success. This means that human skills will be planned, evaluated, and developed to realize the organization’s overall goals. An assumption here is that there exists a relationship between the two: the competence of the individual employee and the overall objectives of the organization. The Norwegian organizational researcher Linda Lai is closely acquainted with the NAFs plans for competence. Lai defines strategic competence management like this:

“Strategic competence management involves planning, implementing and evaluating measures to ensure that the organization and the individual employee has and uses the necessary competence in order to achieve defined goals” (Lai, 2013, p. 14, authors’ translation).

In an interview with Major General Kampenes during the autumn of 2017 that was published in the Norwegian Armed Forces magazine Forsvarets Forum (Forsvarets Forum, 2017), and in the newspaper Aftenposten on October 12, (Bentzrød, 2017), Kampenes speaks out in favor of developing a dedicated organization that will be responsible for all cyber operations. This has also been discussed in the United States, and President Donald Trump has announced that the United States Cyber Defence should be gathered under one command. In the same interview, Kampenes also expressed concern about the NAFCD, in which he briefly states that “*we have little ability to face cyber-attacks*” (authors’ translation) and “*if cyber-attacks are massed against us, we will not last for a long time. We may stop an attack, but we cannot reveal who is attacking*” (Ibid., authors’ translation). This may be linked to a very low level of DD-UN when it comes to handle unforeseen cyber-attacks. In sum, Kampenes believes that there are essentially three types of threats: “*The curious hacker, criminals looking for financial gain, and state actors. The latter may be spying, but it can also be digital attacks*” (Aftenposten, 2017). Such attacks can shut down power plants, black out cities, destroy banking systems, and stop nuclear weapons programs. Kampenes among other things points to examples from Ukraine and Iran in these interviews. Many states are currently struggling with how to understand an ever increasing and interconnected world (Brantley, 2016). Norway is no exception. There exist over 10,000 terrorist websites around the world. This may be seen as an indication that terrorist ideology continues to spread (Weimann, 2015).

The Norwegian Foreign Minister and previous Norwegian Defence Minister, Ine Eriksen Søreide, is clear that the Norwegian Cyber Defence must be strengthened. In a statement to one of Norway’s largest newspapers in the autumn of 2017 after receiving an annual status report from the Norwegian Intelligence Service (NIS) on the threat to Norway, saying: “Norway needs a digital border defense…

…Today’s threat situation is very complex and we must be prepared to protect ourselves from advanced attacks. This includes spying from other countries, preparations for cyber-attacks, and foreign planning of terror on Norwegian soil. This is a serious threat to our safety. Today, almost all communication – 99% – flows in and out of Norway in fiberoptic cables. The NIS does not have its own access to this cable network. In order to solve its statutory duties, the NIS must be able to search this network for information about threats that could harm Norway. But then the service must have access to the relevant data streams – that is, retrieve information where the information is located. And so, it has moved from satellite, where intelligence services have access today, to fiberoptic cables, where they have no access. Norway is currently dependent on information from partners and other countries that have digital border defense systems.
Critical social functions can be under attack for a long time, without us even knowing it. The government is now working on providing the NIS with the tools it needs to protect Norway from digital threats, within strict limits that protect privacy. Should we stop an ever-changing threat, we must act now…
… The state’s main task is to safeguard the security of its inhabitants. It is becoming increasingly demanding. Our new digitized reality gives us enormous opportunities, but at the same time makes us vulnerable in the face of a technological and security policy development that goes very fast. When the threats change, it requires the state to develop new ways we can protect ourselves… ” (Eriksen Søreide, 2017, authors’ translation).

Eriksen Søreide (2017) emphasizes the need for a “digital border defense.” However, we believe that the NAFCD is focusing on the technical expertise and the material side of technology. In a “digital border defense” this is obviously necessary. Eriksen further points out the need for interaction with partners and other countries regarding information. This ties well in with our interpretation that the interaction both within the NAFCD and the interaction that the NAFCD has with other cyber and security organization should be improved. Studies that we have discussed also show that there is a need for more thorough training schemes that emphasize more unforeseen events and challenge the entire organization (Samuelson and Zeckhauser, 1988; Ritov and Baron, 1992; Deibert et al., 2012; Lindsay, 2013; Björck et al., 2015; Sheffi, 2015; Torgersen, 2015; Buchanan, 2016). This is necessary if the NAFCD wants to improve how they cope with the different levels of DD-UN (levels 0–4) in relation to different levels of familiarity, notification, and escalation. This means that focusing on training may aid in moving from level 4 where there are some known characteristics (familiarity), some preparation (notification), and some known warning signs (escalation) to level 0 in the DD-UN model. In level 0, the cyber-attack will be absolutely unknown (familiarity), there is no preparation (notification), and there are no known warning signs (escalation).

The NAFCD has implemented measures so that employees can acquire competencies in their everyday work life. Overall plans are made for the departments, but these are not necessarily linked to the strategic plans. At the top levels, there are guidelines through business plans and through dialog with the defense branches. At the lower levels, it is felt that there are not so many guidelines, other than that soldier competence is a priority. Professional guides do not come from a higher level. Generally speaking, there is a lack of finance, time and available personnel, which are the biggest obstacles for the departments to be able to carry out the skills development and which are necessary for the departments to be able to better manage their assignments. There is a great variety in how and how often courses are evaluated.

Pfeffer and Sutton (2000) have worked on how management can be exercised to ensure learning. Their starting point is that there are people in the organization who hold on to the knowledge. They are concerned with the use of knowledge, i.e., the gap between acknowledging that something should be done and the action required to solve a problem. However, a significant difference is that the organizations and companies that Pfeffer and Sutton investigated were based on geographical aggregation, which is not the case with the NAFCD being spread out both geographically and professionally. Nevertheless, Pfeffer and Sutton’s focus on the individual member of the organization has been important in our approach: “Formal systems can’t store knowledge that isn’t easily described or codified but is nonetheless essential for doing the work, called tacit knowledge” (ibid., p. 19).

### What About the Vision of Competence for a New Era

In the Norwegian Government report 14, subtitled “Competence for a new era,” it is emphasized that the ability “to apply knowledge, to see new solutions and to combine knowledge in new ways is crucial for the tasks the sector is to solve today and to develop and meet future challenges” (Forsvarsdepartementet, 2013, p. 18, authors’ translation). A comprehensive theme in the report is the importance of the leaders’ strategic competence in this process. The text in the report contains few detailed instructions on how this competence can be safeguarded or exercised. According to Lai (2004), competence is to possess the necessary knowledge, skills, abilities, and attitudes to master tasks and achieve goals. Competence is acquired through education, training, work experience and ongoing competence development at the workplace and through different types of continuing education (Saga Corporate Advisors, 2011). Whichever definition of competence one chooses, knowledge, skills, and abilities will be core components of the competence concept at the individual level (Moland et al., 2010). In order to develop the level of competence of the employees and the different organizations, there has to be a conscious reasoning and action concerning competence activities. We call this thinking SCL. SCL consists of three main elements: planning, implementation and evaluation (Lai, 2004; Moland et al., 2010).

At the same time, basic skills, technology systems and interaction routines at the NAFCD need to be practiced so that this is in place. Such exercises must be done on a regular basis and must be included in comprehensive competence plans. At the same time, it is imperative that the strategic leadership follows up, are updated regularly on new forms of technology leadership, and are involved in both their own competence development and in the strategic competence development for their defense branch, that is, the NAFCD and the other defense branches and units in the nation. Otherwise, the NAFCD may suffer from what Lai (2004) calls *incongruence competence*, or incompetence.

### Training for the Unforeseen

The different components of the DD-UN as a training model (see **Figure 2**) for cyber competence and the unforeseen reveals that the NAFCD will have to implement different competence development within training, leadership and scenarios. On the other hand, the cyber domain has a highly dynamic and flexible character that creates a number of critical challenges as to how cyber should be approached militarily. This may be exemplified by the notion that cyber is itself in rapid development. In addition, cyber weapons are also distinguished by a rapid development (Rid and McBurney, 2012).

In light of the SD-UN model (see **Figure 1**) there will be a need for more training on events that challenges the lower competence structures, that is, the competence unforeseen factors and basic capacities, for both levels of participants in our investigation.

Our results reveal that there existed a different understanding of the SCL between the high-level and the low-level participants. This may affect the interaction between leaders at different high levels and the lower levels. This might also affect the interaction with other security or emergency departments, and may hamper cross-cultural training with for instance the Norwegian police or other civilian cyber units. Training on interaction will have to be conducted under daily supervision and simulated cyber-attacks will be necessary.

For those on a lower level, primarily the executive personnel with a technological expertise, there is a different competence need. There is here a need for training that combines technology as a part of the learning and change element in the SD-UN model with for instance warning signs. This can be accomplished by inserting hidden “worms” or other ambiguous information that does not appear to be a threat, but are a part of scenario-simulations, preferably not pre-warned or announced as a part of the training on improvisation. This will enhance the level of competence unforeseen factors.

However, good planning, costly equipment and software, and time-consuming preparations are required. It may be worth it. For the high-level leaders, flexibility in decision-making will need to be exercised, increasing the competence unforeseen factors for this group. The low-level employees and technology experts must be allowed to make meaningful decisions based on first-hand information. The low-level employees should also gain more insight into what unforeseen cyber-attacks may involve, where the core is that new events rarely resemble past and known events. This will probably challenge the decision-making basis of the high-level leaders, and dilemma training under such conditions will be necessary. Khooshabeh and Lucas (2018) has pointed out that the cyber operators (equivalent to low-level participants in our study) have more domain knowledge than the leaders (equivalent to high-level participants in our study). This might affect the social dynamic between the cyber operators and leaders. Increased unforeseen cyber competence so that the NAFCD raises its competence status as measured in the DD-UN levels (see **Table 1**) at all levels of organization will require systematic efforts to develop competence skills and training plans. This will also include the development of scenarios and custom technology, which can be changed on a continuous basis. Thus, this is part of the strategic plans for the entire organization – also in terms of budgeting.

Overall, there will be a need for more targeted training on professional/technical, cognitive and interaction-oriented challenges at all levels in the NAFCD. This is in line with Spiro et al. (1992) and their thinking around cognitive flexibility. Also, cross-sectional training between different organizations in order to face unforeseen events will be important. The exercise tasks should be subjected to a through principle of training on: (1) in fellowship to extract knowledge from disorder in information and surroundings, (2) competence exchange under loss of control, and (3) flow by chaos and creating room for surprise (Torgersen, 2018).

## Conclusion

We have in this study used a grounded theory method to investigate how well prepared the NAFCD is for SCL and competence development for future competence needs, especially in relation to the unforeseen.

A national digital border defense also involves interaction with other nations, thus challenging the competence and communication chains across borders and technological barriers. Not only is new technology needed, but also a new education and comprehensive SCL, which is constantly changing – and preferably a fraction ahead of the enemy. A conclusion drawn from our data is that education programs that are designed to enhance SCL also must support an improved interaction between different involved actors, state or non-state. This might be a severe challenge, as the educational-psychological status regarding a digital border defense within the NAFCD might be reluctant to change. On the other hand, there are some positive changes issued from a higher level that might take care of these challenges. The Norwegian Ministry of Defence has issued a new security law (Forsvarsdepartementet, 2017). The new law explicitly declares who is responsible for preventive security work. Each ministry will be responsible for the preventive security work within their respective sectors. The new law is also adapted toward the technological development and is meant to secure electronic documents. The new law will thus aid in identifying which changes in the educational-psychological status that are needed for the NAFCD in the future. This may render the Digital Border Defense more effective. A useful stepping stone for the NAFCD would be to converge on what SCL as a concept is, and how it should be used to enhance ongoing education and competence development in order to face unforeseen events.

An interesting future research direction would be to revisit the NAFCD at a later date, and then to replicate our study, to investigate if there has been any development of the NAFCD since conducting our study. A similar study can also be conducted in other branches of the NAF and also in other emergency preparedness agencies to reveal how well they are equipped for the unforeseen. In addition to this, studies of compliance issues and articulation errors within the ICT businesses with regard to unforeseen issues are another possibility. There is also a possibility of studying compliance issues and articulation errors between public actors and private actors.

### Possible Limitations

A limitation in our study is that the number of participants was quite low. This was caused by the relatively low number of possible participants in the NAFCD. This also meant that it was not possible to perform any statistical calculations. The external validity of our qualitative results is also quite low. Another possible limitation is that the different leadership and management levels in the NAFCD could be considered as quite homogeneous groups. This is due to the fact that all managers and leaders have been through the same type of selection into the NAF. As such, there might not be such a large difference between the low-level and the high-level group as we think there is. Also, the selection of participants was made to be within economical limits and time limits when it came to for instance traveling to participants. This means that we may have missed certain participants that would have given different answers to the questions posed in the study. A final possible limitation could be that we conducted a study of a part of our own organization, that is, the NAF. As such, we may have been less objective than researchers from outside the NAF might have been when investigating the same research problem.

## Author Contributions

G-ET and OB designed the study, developed and drew figures and models. G-ET and OB also wrote the manuscript, analyzed the data, revised the manuscript, and approved the final version to be published.

## Conflict of Interest Statement

The authors declare that the research was conducted in the absence of any commercial or financial relationships that could be construed as a potential conflict of interest. The reviewer BK declared a shared affiliation, with no collaboration, with the authors to the handling Editor.
